# Successful Management of Iatrogenic Cranial Pseudomeningocele With Subgaleal Shunt

**DOI:** 10.7759/cureus.34513

**Published:** 2023-02-01

**Authors:** Buqing Liang, Yilu Zhang, Kristopher Lyon, Jose M Soto, Anthony Nguyen, Jason H Huang, Dongxia Feng

**Affiliations:** 1 Neurosurgery, Baylor Scott & White Health, Temple, USA

**Keywords:** pleural shunt, peritoneal shunt, cerebrospinal fluid (csf), subgaleal, shunt, pseudomeningocele

## Abstract

Iatrogenic pseudomeningocele is a common complication of cranial surgeries. However, there are no evidence-based guidelines on how to manage this condition. We report two cases of iatrogenic postoperative cranial pseudomeningocele that failed conservative management including compressive head dressing. Subgaleal shunt placement was utilized with successful resolution in both cases. We postulate that subgaleal shunt placement may be an effective method in the management of iatrogenic subgaleal pseudomeningocele.

## Introduction

Iatrogenic pseudomeningocele is a common complication in patients who undergo cranial and spinal surgeries, with an incidence rate greater than 40% [1-3]. Presently, there are no evidence-based guidelines addressing the management of this condition, and both conservative management and surgical intervention are routinely employed. One possible surgical option is the placement of a subgaleal shunt although the literature regarding this method is scarce. Herein, we report two such cases that were successfully managed by subgaleal shunting.

## Case presentation

Case 1

**Figure 1:**
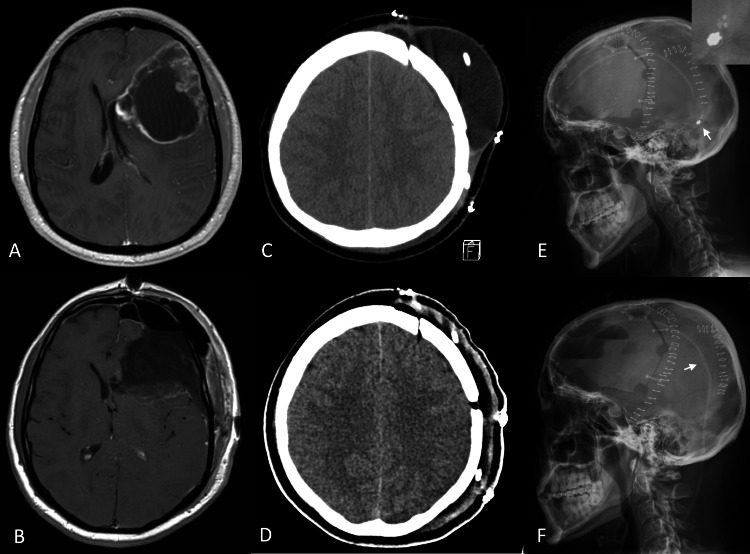
Asymptomatic pseudomeningocele in Case 1 (A) Pre-operative gadolinium-enhanced T1-weighted magnetic resonance imaging (MRI) of the brain revealed a peripherally enhancing mass in the left frontal lobe consistent with a neoplasm. (B) Post-operative gadolinium-enhanced T1-weighted MRI of the brain revealed gross total resection of the lesion (pathology: World Health Organization grade IV glioma). (C) Computed tomography (CT) scan of the head without contrast revealed a large subgaleal pseudomeningocele at the surgical site. (D) CT demonstrating total resolution of the pseudomeningocele at one-week clinic visit after the shunt revision with removal of the shunt valve and direct connection of the proximal and distal catheters. (E) X-ray of the skull demonstrating the Strata II (Medtronic, Minneapolis, Minnesota, U.S.A.) setting at a minimum of 0.5 (white arrow, enlarged figure in right upper inset picture); at this setting, the patient’s pseudomeningocele persisted. (F) Post-revision x-ray of the skull demonstrating proximal and distal tubes connected directly (white arrow); the pseudomeningocele resolved following this revision.

A 40-year-old otherwise healthy male presented with headache and confusion and was found to have a large left frontal intra-axial lesion. He underwent left frontal craniotomy and lesion resection. The dura was then closed water-tight with onlay Duragen and Duraseal. The histopathologic diagnosis was World Health Organization (WHO) grade IV glioma, and he was discharged home on postoperative day (POD) 4 without complications. However, he developed an asymptomatic pseudomeningocele that was refractory to compressive head dressing. He underwent subgaleal-peritoneal shunt placement three weeks after the first operation. A Strata II (Medtronic, Minneapolis, Minnesota, U.S.A.) valve was used and initially set at 1.5. The shunt was subsequently programmed to its minimal setting of 0.5 in the clinic for maximal drainage. Despite this, in combination with continuous compressive head dressing, the pseudomeningocele persisted. Six weeks after his first surgery, the patient underwent a third operation in which the valve was removed, and the proximal and distal catheters were connected directly with a connector. At this one-week postoperative visit, the patient’s pseudomeningocele had resolved (Figures 1A-1F).

Case 2

**Figure 2:**
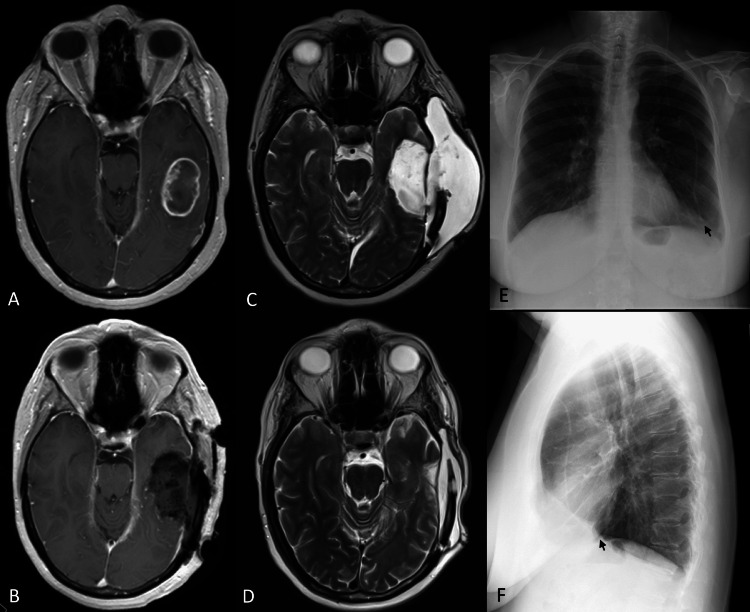
Pseudomeningocele resolved without compressive head dressing (A) Pre-operative gadolinium-enhanced T1-weighted magnetic resonance imaging (MRI) of the brain revealed a ring-enhancing mass at the left temporal lobe consistent with a neoplasm. (B) Post-operative gadolinium-enhanced T1-weighted MRI revealed a gross total resection of the lesion (pathology: World Health Organization grade IV glioma). (C) T2-weighted MRI revealed a sizeable pseudomeningocele. (D) T2-weighted MRI obtained following subgaleal-pleural shunt placement revealed resolution of the pseudomeningocele. (E, F) Anteroposterior and lateral view chest X-rays demonstrating the distal end of the catheter ending in the left pleural space (black arrow).

A 59-year-old female with a past medical history of endometrial cancer status post hysterectomy, chemotherapy, and radiation therapy presented with two episodes of generalized seizures and was found to have a left temporal intra-axial lesion. She underwent left temporal craniotomy and lesion resection. The dura was then closed water-tight with onlay Duragen and Duraseal. Histopathologic diagnosis was WHO grade IV glioma. She was discharged home on POD 7 without complication. However, she then developed an asymptomatic pseudomeningocele which was refractory to compressive head dressing. Considering the patient’s abdominal surgical history of prior cholecystectomy, Cesarean section x2, and hysterectomy, the decision was made to proceed with a left subgaleal-pleural shunt placement with the assistance of thoracic surgery one month after her initial craniotomy. A Strata II (Medtronic, Minneapolis, Minnesota, U.S.A.) valve was used and initially set at 1.5 which was subsequently adjusted to 0.5 in the clinic. The pseudomeningocele gradually improved and resolved without compressive head dressing (Figures 2A-2F).

## Discussion

Iatrogenic pseudomeningocele is a common complication after cranial or spinal surgeries due to dural defects and the resultant accumulation of extradural cerebrospinal fluid (CSF) at the surgical site [1,2]. Pseudomeningocele of the skull can be asymptomatic, resulting in only an unaesthetic appearance. They can also result in severe complications, leading to wound dehiscence, meningitis, CSF fistula, intracranial hypotension, or even death [1]. Conservative management includes observation, head elevation, fluid aspiration, compressive head wrapping, or medications that decrease the production of CSF. Less invasive surgical options include temporary diversion by the placement of a lumbar drain or external ventriculostomy drain [4]. The majority of cases resolve without sequelae [1-5]. However, for persistent or recurrent pseudomeningocele, surgical intervention is sometimes warranted. Open surgery for dural defect repair and shunt placement has both been utilized. Different shunting techniques have been reported. In cranial cases, ventricular catheter placement is preferred for cases with hydrocephalus. Intrathecal catheter placement via a lumbar puncture or direct placement inside the pseudomeningocele are other options available for spinal pseudomeningoceles; the latter is described above for cranial pseudomeningoceles. Programmable valves are commonly used to connect the proximal and distal parts of the shunting system. Compartments for placement of the distal catheter most commonly include the peritoneal cavity, pleural space, and right atrium. Another option described for spinal pseudomeningocele is the epidural blood patch, which has been reported in the literature [6].

In case 1, the patient did not have headache, nausea, vomiting, hydrocephalus, or transependymal flow on imaging, indicating that intracranial pressure was normal. Therefore, even with the minimum shunt valve setting, the drainage was ineffective. After direct connection of the proximal and distal catheters, with the siphon effect, the subgaleal fluid collection subsided and resolved. This technique has also been effective in another case report [7]. One potential concern is retrograde fluid flow from peritoneal cavity into the subgaleal space, with a theoretically increased risk of meningitis. However, intraperitoneal space is sterile unless there is a bowel perforation, bile leakage, or other infectious intra-abdominal pathology. To further reduce the risk of complications, the patient was educated on the avoidance of sudden position changes from recumbence to standing, lowering the risk of dramatic drop in intracranial pressure.

For patients with multiple abdominal surgeries or any other contraindications to peritoneal shunt placement, the thoracic cavity is an alternative destination, as described in our second case. Considering less frequent pleural shunt placement in practice, cardiothoracic surgeons may need to be consulted for operative assistance. However, the thoracic cavity is occupied by the lungs, and its capacity for fluid absorption is limited compared to the peritoneal cavity [8]. With pleural effusions, lung expansion can be restricted, leading to hypoxemia. Close postoperative monitoring can aid in prompt identification and treatment of pleural effusion. Our patient was monitored in the intensive care unit for 48 hours and underwent postoperative imaging consisting of serial chest x-rays and a computed tomography (CT) scan of the chest. She was discharged without evidence of accumulation of pleural effusion or shortness of breath.

The timing of surgery is mainly surgeon dependent. According to Tu et al. [1], surgical intervention would be attempted after an average of 7-14 days of conservative management. In our cases, subgaleal shunt was placed after 2-3 weeks of a failed trial of conservative management. Since most cranial pseudomeningoceles resolve spontaneously [5], Tu et al. [1] surmise that early surgery is not indicated.

Like any other shunt system, potential malfunction can occur. Obstruction, migration, infection, bowel perforation, pseudocyst formation, etc. have all been reported [7-9]. Long-term follow-up is needed in any patients with a shunt to monitor for both short-term and delayed complications.

Regardless of the efficacy of treatments for pseudomeningocele, prevention is ideal. Techniques to reduce the risk of pseudomeningocele development include water-tight closure, duroplasty with grafts, and utilization of tissue glues and blood patch have all been proposed [6,10]. However, if ependyma is transgressed during surgery, “high-flow” CSF leak is encountered [11]. In our two cases, both tumors were abutting the lateral ventricles (Figures 1A, 2A) which were inevitably entered in order to achieve gross total resection, thus increasing the risk of pseudomeningocele. Despite water-tight dural closure, utilization of a Duragen onlay, and Duraseal, pseudomeningocele still developed.

This study is a retrospective single-institute case report which limits its ability for generalization. The utility of this technique in cases with infratentorial pseudomeningocele also needs to be explored. Additional prospective studies with larger sample sizes are necessary to determine the efficacy of this technique in the treatment of pseudomeningocele.

## Conclusions

Iatrogenic pseudomeningocele is a common complication of cranial surgeries. For cases refractory to medical management such as observation, head elevation, fluid aspiration, compressive head wrapping, or medications, subgaleal shunt placement may be a safe and effective treatment option.
